# Modulation of the Host Nuclear Compartment by *Trypanosoma cruzi* Uncovers Effects on Host Transcription and Splicing Machinery

**DOI:** 10.3389/fcimb.2021.718028

**Published:** 2021-10-19

**Authors:** Camila Gachet-Castro, Felipe Freitas-Castro, Raul Alexander Gonzáles-Córdova, Carol Kobori da Fonseca, Marcelo Damário Gomes, Hellen Cristina Ishikawa-Ankerhold, Munira Muhammad Abdel Baqui

**Affiliations:** ^1^ Department of Cellular and Molecular Biology and Pathogenic Bioagents, Ribeirão Preto Medical School, University of São Paulo, Ribeirão Preto, Brazil; ^2^ Department of Biochemistry and Immunology, Ribeirão Preto Medical School, Ribeirão Preto Medical School, University of São Paulo, Ribeirão Preto, Brazil; ^3^ Department of Internal Medicine I, Ludwig-Maximilians-University of Munich, Munich, Germany

**Keywords:** host-pathogen interaction, trypanosomatid, nucleus, ribonucleoproteins, splicing factors, RNAPII, U2AF35

## Abstract

Host manipulation is a common strategy for invading pathogens. *Trypanosoma cruzi*, the causative agent of Chagas Disease, lives intracellularly within host cells. During infection, parasite-associated modifications occur to the host cell metabolism and morphology. However, little is known about the effect of *T. cruzi* infection on the host cell nucleus and nuclear functionality. Here, we show that *T. cruzi* can modulate host transcription and splicing machinery in non-professional phagocytic cells during infection. We found that *T. cruzi* regulates host RNA polymerase II (RNAPII) in a time-dependent manner, resulting in a drastic decrease in RNAPII activity. Furthermore, host cell ribonucleoproteins associated with mRNA transcription (hnRNPA1 and AB2) are downregulated concurrently. We reasoned that *T. cruzi* may hijack the host U2AF35 auxiliary factor, a key regulator for RNA processing, as a strategy to affect the splicing machinery activities directly. In support of our hypothesis, we carried out *in vivo* splicing assays using an adenovirus E1A pre-mRNA splicing reporter, showing that intracellular *T. cruzi* directly modulates the host cells by appropriating U2AF35. For the first time, our results provide evidence of a complex and intimate molecular relationship between *T. cruzi* and the host cell nucleus during infection.

## Highlights

*Trypanosoma cruzi* modulates host transcription and splicing machinery by downregulation host ribonucleoproteins and critical splicing factors required for RNA processing.

## Introduction

*Trypanosoma cruzi* is the etiological agent of American trypanosomiasis (Chagas Disease; CD), a debilitating and Neglected Tropical Disease (NTD) responsible for ~ 10,000 deaths annually (World Health Organization, 2021). CD primarily affects individuals in Latin America, costing ~ $ 1.2 billion of productivity per year; however, an estimated 8 million individuals worldwide are infected with *T. cruzi*, attributed to increasing international mobility. Many affected individuals develop severe cardiac or digestive pathology (Pérez-Molina and Molina, 2018); however, treatment for American trypanosomiasis is limited, costly, and requires hospitalization and drug therapy for symptom management (Moncayo and Silveira, 2009). In an added complication, the therapeutic agents available for the treatment of CD are effective only in the acute phase of the infection and often cause toxicity to the patient, highlighting a need to understand better the pathogen-host interaction in the search for new therapeutic targets (Cancado, 2002; Sánchez-Valdéz and Padilla, 2019).

Infection by *T. cruzi* triggers biochemical and morphological changes in both host and pathogen cells, including a series of cellular signaling processes culminating in the recruitment of lysosomes to the host cell plasma membrane, subsequently promoting the formation of a Parasitophorous Vacuole (PV) and internalization of *T. cruzi* in the target cell (Burleigh, 2005; Yoshida, 2006; De Souza et al., 2010; Maeda et al., 2012). During its intracellular cycle, within the host cytoplasm, the internalized trypanosomes move inward towards to the host cell nucleus (Zhao et al., 2013), suggesting these parasites may interact with the host nucleus. Though as to why *T. cruzi* localizes here, and if this parasite can alter nuclear organization or host gene expression is unknown, though recent reports suggest that *T. cruzi* does interfere in central host cell metabolism (Shah-Simpson et al., 2017; Lentini et al., 2018). Manipulation of the host’s nuclear compartment and epigenome has been reported in other intracellular pathogens (Paschos and Allday, 2010; Hari Dass and Vyas, 2014; Sinclair et al., 2014; Robert Mcmaster et al., 2016). For instance, intracellular *Leishmania*, a related kinetoplastid, which causes Leishmaniasis, secretes the surface protein GP63 early in infection, which can alter the nuclear envelope of the host macrophage, promoting nuclear physiology in favor of parasite virulence (Isnard et al., 2015).

Furthermore, the apicomplexan parasite *Toxoplasma gondii* secretes molecules, which work as molecular switches and alter the host’s transcriptional activity (Melo et al., 2013; Hakimi and Bougdour, 2015). Recently studies have revealed that schizont from the surface structures of *Theileria* is strongly associated with the nuclear membrane of their host cells, being close to the nuclear pores in infected T and B cells and macrophages (Huber et al., 2020). Thus, given the proximity of *T. cruzi* to the host cell nucleus, we reasoned that this parasite might also be capable of exerting alterations to host gene expression that promote parasite survival.

Our data shows that intracellular *T. cruzi*, which replicates within the host cell cytoplasm, can manipulate the host transcription program and host splicing machinery. This effect is time-dependent and is associated with parasite persistence in the host cell. Furthermore, we observed increased host cell nuclear membrane distortion in host cells with the most nuclear proximal parasites suggesting parasite positioning correlating with parasite proximity to the host nucleus suggesting parasite positioning can induce deformation in the nuclear envelope (NE) even in the absence of nuclear invasion. We propose that this strategic localization reflects the first signaling step during host machinery manipulation, resulting in transcription modulation and corresponding signaling pathway modifications. Remarkably, we found that *T. cruzi* hijacks the mammalian splicing factor U2 snRNP auxiliary factor 1 (U2AF35), affecting the splicing of host cell transcripts. Consequently, we detected downregulation of host cell proteins associated with mRNA transcription, processing, and transport machinery at 11–12 hours post-infection (hpi). Disturbing these processes appears to be fundamental for the intracellular development of *T. cruzi* amastigotes. Taken together, we reveal a novel interaction between *T. cruzi* and the host cell nucleus during infection. We show that *T. cruzi* is capable of moderating host transcription and splicing *via* the sequestration of a host splicing factor U2AF35, thereby faltering the environment of the host cell in favor of parasite survival.

## Material and methods

### Parasites

*Trypanosoma cruzi* (strain Y or Tulahuen), used in the present study, was routinely maintained in Balb/c mice by repeating passages to keep its virulence. Promastigote *Leishmania major* (MRHO/SU/59/P) strain LV39, from axenic culture, was maintained at 26°C in M199 medium supplemented as previously described (Coderre et al., 1983). *Trypanosoma cruzi* strain Y in epimastigote form from axenic culture was maintained at 28°C in LIT medium supplemented as described (Camargo, 1964).

### Cell Culture and Infections

LLC-MK2 (American Type Culture Collection (ATCC), cat. CCL-7) and THP-1 (Cell Bank of Rio de Janeiro (BCRJ), code: 0234) were maintained in culture at 37°C in 5% CO_2_ in RPMI medium (Gibco, USA) supplemented with 10% heat-inactivated fetal bovine serum (FBS) (Gibco, USA) and antibiotics (Penicillin 100 U/ml and Streptomycin 100 µg/ml, Sigma, USA). THP-1 cells were differentiated to macrophages with 50 ng/mL of Phorbol-12-myristate-13-acetate (PMA – Sigma) and incubated for 48h at 37°C with 5% CO2 before infection. HeLa cells (ATCC, strain CC1) were maintained in culture at 37°C in 5% CO_2_ in Dulbecco’s modified Eagle’s medium (DMEM) (Gibco, USA) supplemented with 10% heat-inactivated FBS (Gibco, USA) and antibiotics: Penicillin 100 U/ml and Streptomycin 100 µg/ml. LLC-MK2 cells were infected with *Trypanosoma cruzi*, collected directly from Balb/c mice blood. After two passages in cell monolayers, trypomastigotes were collected, and new plates were reinfected in the multiplicity of infection (MOI 10:1) to perform experiments. *L. major* obtained from stationary-phase culture (5 days after seeding) was added in the same proportion as *T. cruzi* to the experiment after resuspension in complete RPMI media.

### Antibodies

Antibodies used in this study include anti-TcFAZ antibody against a *T. cruzi* protein localized at the FAZ (prepared in house, manuscript in preparation), as well commercial antibodies as follow: Anti-SC35 (#S4045 - mouse), anti-GAPDH (#PLA0125 - rabbit), anti-U2AF35 (#SAB1300700 - rabbit), anti-hnRNPA1 (#R4528 - mouse), hnRNPA2B1 (#R4653 - mouse), and were from Sigma, USA. Other commercial antibodies included anti-RNA polymerase II - 8WG16, H5 and H14 (#MPY-127R - mouse, Covance, USA), anti-mouse IgM-HRP (#sc-2064 - goat, Santa Cruz, USA), biotin IgM conjugated secondary antibody (#553406 – rat, BD, USA), Streptavidin-HRP (#554066, BD).

### Immunofluorescence Assay

LLC-MK2, THP-1 cells, or HeLa cells were settled for 16h in 24-well plates containing UV sterilized coverslips and then infected from 0–24h with *T. cruzi* (MOI 10:1). Then, cells were washed twice with PBS, fixed for 10 min with PBS/paraformaldehyde 2%, and permeabilized for 10 min at RT with Igepal 0.2% in PBS. Coverslips were blocked with PBS/BSA 2% solution and incubated with primary antibodies for 30 min at RT. Cells were labeled with goat anti-mouse IgG antibodies conjugated to Alexa Fluor 488 (Life Technologies, USA), and actin was stained with Rhodamine phalloidin (Life Technologies, USA). The experiment was finalized with ProLong Gold antifade reagent containing DAPI (Thermo Fisher Scientific, USA). The confocal image stacks were acquired in TCS-SP5 Leica Confocal Microscope, with a 63X oil immersion objective and detected with Photomultiplier tube (PMT) and Hybrid detector (HyD) from the Multiuser Laboratory of Confocal Microscopy, Department of Cell and Molecular Biology and Pathogenic Bioagents, Ribeirão Preto Medical School, University of São Paulo (FMRP/USP) and with a DMI 4000B Epifluorescence microscope (Biochemistry and Immunology Department, FMRP/USP. Super-resolution images were acquired using a LSM880 laser scanning confocal microscope, equipped with Airyscan super-resolution module from ZEISS, with the PlanApo 63X oil immersion 1.4 objective, and analyzed using the ZEN software at the Imaging Core Facility, Walter Brendel Center of Experimental Medicine-LMU, Munich/Germany. Unless stated, all reagents were purchased from Sigma-Aldrich.

### Total Cell Lysates

LLC-MK2, HeLa, and THP-1 cells were settled in six-well plates until reaching confluency. The cells were infected with *T. cruzi* (MOI 10:1) at 1–24h. Adherent cells were washed five times with PBS to remove extracellular parasites. Then cells were treated with trypsin, collected, and centrifuged at 2500x*g*/4°C/5 min. The cell pellet was incubated with radioimmunoprecipitation assay (RIPA) buffer [20 mM Tris, pH 7.2, 150 mM NaCl, 1% Triton X-100, 1% sodium deoxycholate, 0.1% sodium dodecyl sulphate (SDS)] containing protease and phosphatase inhibitors (Moreira et al., 2017). Tubes were kept on ice for 10 minutes, then the lysates were mixed and centrifuged at 14000x*g* for 10min to pellet cell debris. Supernatants were collected, and the total protein was measured *via* spectrophotometry at 280nm. Samples were resuspended in sample buffer for analysis by SDS-PAGE. Unless stated, all reagents were purchased from Sigma-Aldrich.

### Immunoblotting Assay

Total cell lysate (approximately 100μg) was resolved by SDS-PAGE (8% or 12%) and transferred to nitrocellulose membranes in semi-dry blot apparatus (Transfer Blot SD, Bio-Rad) using transfer buffer without methanol (Moreira et al., 2017). Membranes were blocked with 5% skimmed milk in TBS/T solution (50 mM Tris, pH 8, 150 mM NaCl, Tween 0.05%). Membranes were washed three times with TBS/T solution and incubated at RT with the primary antibody diluted in TBS/T (anti-RNA polymerase II 8W, H5 1:500 and H14 1:500; hnRNPA1 1:3000; hnRNP A2/B1 1:1000; GAPDH 1: 20000; and anti-U2AF35 1:500). Then, membranes were incubated either with goat anti-mouse IgG or IgM-peroxidase-conjugated antibodies, respectively, goat anti-rabbit IgG peroxidase-conjugated antibodies, IgM-Biotin and Streptavidin peroxidase-conjugated antibodies, and the signal was detected with enhanced chemiluminescence ECL Prime Western Blotting Detection Reagent (GE Healthcare, USA) according to the manufacturer’s instructions. Images were acquired in Image Quant LAS4000 (GE, USA), Ribeirão Preto Medical School, University of São Paulo (FMRP/USP).

### RNA Labeling

Adherent cells were fixed in pre-chilled methanol (Sigma-Aldrich, USA) at -20°C for 10 min and washed with PBS at RT. According to the manufacturer’s instructions, total RNA was labeled using 500 nM Syto RNA Select (Molecular Probes, USA). Coverslips were mounted using cell ProLong antifade with DAPI. Images were acquired using an Epifluorescence microscope Leica (DMI 4000B) and a Confocal Microscope Leica (TCS-SP5).

### Image Measurements

Fluorescence microscopy images were analyzed by ImageJ software, version 1.49v (Rasband, W.S., ImageJ, U. S. National Institutes of Health, Bethesda, Maryland, USA, http://imagej.nih.gov/ij/) to evaluate nuclear-stained host cells. The fluorescence intensity of the nuclear proteins acquired in epifluorescence microscope was measured using the ROI management tool and subtracting the background. Total cell-stained RNA was analyzed in Total Corrected Fluorescence Cell (CTCF). Subsequently, graphs were plotted with the fluorescence average values using GraphPad Prism 5 program (GraphPad Software, Inc., San Diego, CA). The quantifications were done in cells that contained parasites at the analyzed time of infection. All images were acquired using the same light or laser intensity, gain, brightness, and contrast standards. The images shown in the figures have been brightness and contrast modified for better viewing using Adobe Photoshop CC software (version 2017.1.0). All analyses were performed using original, unmodified images.

### Electron Microscopy

For Transmission Electron Microscopy (TEM), cell cultures (control and infected cells) were fixed by immersion in 2% glutaraldehyde and 2% paraformaldehyde in 0.1M cacodylate (EM Sciences, USA) buffer, post-fixed in 1% osmium tetroxide (EMS, USA), and stained in a block with 2% uranium acetate (EM Sciences, USA) in 0.1M sodium acetate (pH5.0). Samples were dehydrated in ethanol and afterward with propylene oxide and were embedded in resin – Embed 812 (EM Sciences, USA). Ultrathin sections were collected on pioloform (Ted Pella, USA) and carbon-coated single-slot grids and then contrasted with uranyl acetate and lead citrate (Moreira et al., 2017). After embedding and sectioning, the images were acquired at 120 kV using a Tecnai G2 electron microscope (LCMS, Laboratory of Cellular and Molecular Ultrastructure, Ribeirão Preto Medical School, University of São Paulo, FMRP/USP. All reagents not specified above were purchased from Sigma-Aldrich. Scanning Electron Microscopy (SEM) was performed as described elsewhere (Moreira et al., 2017; Sant’Anna et al., 2005). Images were acquired using Jeol JSM-6610 LV microscope (LMME – Multiuser Electron Microscopy Laboratory, FMRP/USP).

### Reverse Transcription-Quantitative PCR

LLC-MK2 cells (NI, 4-24h) were lysed using TRIzol reagent (Invitrogen, USA). Prepared samples were applied directly to the Zymo-Spin Column (Zymo, USA) for RNA purification, which was done according to the manufacturer’s instructions. The extracted RNA was treated with TURBO™ DNase (Thermo Fisher Scientific, USA). Next, total RNA (± 700ng per sample) was transcribed into cDNA using the High-Capacity cDNA Reverse Transcription Kit (Applied Biosystems, USA). Reactions were conducted in technical and biological triplicates using SYBR Green (Applied Biosystems, USA). The optimization of the RT-qPCR was done according to the manufacturer’s instructions (Applied Biosystems User Bulletin 2, applied to the SYBR-Green I core reagent protocol). The following specific primers were used for quantification:

U2AF35

forward 5’-CTGCTGCCGTCAGTATGAGA-3’

reverse 5’-CTCTGGAAATGGGCTTCAAA-3’;

hnRNPA1 (Boukakis et al., 2010)

forward 5’-CCAGAGAAGATTCTCAAAGACC-3’

reverse 5’-CTTCAGTGTCTTCTTTAATGCC-3’;

hnRNPA2B1 (Boukakis et al., 2010)

forward 5’-AGCTTTGAAACCACAGAAGAA-3’

reverse 5’-TTGATCTTTTGCTTGCAGGA-3’;

GAPDH (Galiveti et al., 2010)

forward 5’-CTGGTAAAGTGGATATTGTTGCCAT-3’

reverse 5’- TGGAATCATATTGGAACATGTAAACC-3’.

Data were analyzed according to the 2^-ΔΔCt^ method using GAPDH for normalization. Uninfected cells were used as calibrator condition according to the strategy previously described (Schmittgen and Livak, 2008). To evaluate target amplification efficiency, a standard curve was generated using 4-fold serial dilutions of cDNA. Data correspond to the means and standard deviations ( ± SD) from 3 independent experiments (n=3), analyzed using GraphPad (Prism 5). Statistics were performed using One-way ANOVA with *post hoc* Tukey tests. Data were considered significant if p<0.05.

### *In Vivo* Splicing Assay

*In vivo* splicing assay was performed as described elsewhere (Bressan et al., 2009). Briefly, 4x10^5^ LLC-MK2 cells/well were transfected with 2µg of splicing reporter minigene E1A (pMTE1A), or co-transfected with 2µg of E1A and 0,1 µg of EGFP or with 2µg of E1A and 0,1 µg of U2AF35-EGFP using Lipofectamine 2000 (Thermo Fisher, USA) according to manufacturer’s specifications. After 24h of plasmid expression, the media was replaced, and cells were infected with *T. cruzi* at different times (0, 6, 11, 12, and 13h). After that, cell cultures were washed twice with PBS (Sigma-Aldrich, USA) to remove non-internalized parasites, and total RNA was obtained using TRIzol reagent (Thermo Fisher, USA) according to the protocol recommended by the company. Two µg of RNA was used for Reverse Transcriptase (Thermo Fisher, USA) reaction. The PCRs were performed with primer pairs for E1A, actin, and GAPDH and were carried out using conditions previously described (Bressan et al., 2009). PCR products were analyzed on 2 or 3% agarose gel, stained with ethidium bromide, and Images were acquired in Image Quant LAS4000 (GE, USA). The PCR band abundance was calculated using ImageJ (Schindelin et al., 2012). β-actin or GAPDH bands were used as the baseline control for normalization. To calculate the percentage of each isoform band, we consider all isoform bands as 100% and calculate the proportion of each isoform to plot the graph (Mccloy et al., 2014).

### Blast Analysis

The immunogen sequence of human U2AF35 antibody (Sigma-Aldrich, USA –SAB1300700) was used to search for sequence similarities in trypanosomatids on TritrypDB (http://tritrypdb.org/tritrypdb/) using BLASTp default parameters.

### Cloning and Transfection Assay

To obtain the U2AF35-EGFP construct, we use LLC-MK2 cDNA as a template and U2AF35 specific primers (forward: 5’-GGATCCTCAGAATCGCCCAGATCTTT-3’ and reverse: 5’-CTCGAGTTGCGGAGTATCTGGCCTCCA-3’). Next, we cloned in a frame the U2AF35-EGFP sequence into a pEGFP-C1 plasmid (Clontech, USA) and confirmed the sequence by sequencing (ABI 3500 XL, Applied Biosystems, USA). LLC-MK2 cell line was transfected with 0,1 µg U2AF35-EGFP or 0,1 µg empty-EGFP vector using Lipofectamine 2000 (Thermo Fisher Scientific, USA), according to the manufacturer’s recommendations. Then, LLC-MK2 transfected cells were infected with *T. cruzi* and fixed with PBS/paraformaldehyde 2% for 10 min, then stained with DAPI and Rhodamine phalloidin as described above.

### Ethics Statement

The care of the mice followed the institutional guidelines on ethics in animal experiments; approved by CETEA (Comissão de Ética em Experimentação Animal da Faculdade de Medicina de Ribeirão Preto, approved protocol number 121/2016). CETEA follow the Brazilian national guidelines recommended by CONCEA (Conselho Nacional de Controle em Experimentação Animal). For euthanasia, the mice were treated with ketamine and xylazine (50 mg/kg and 10 mg/kg, respectively) by intravenous injection

### Statistical Analysis

The different times of *T. cruzi* infected cells were compared to non-infected cells using either one-way analysis of variance (ANOVA) and Bonferroni Multiple Comparison test or non-parametric t-test (Mann-Whitney). All statistical analyses were performed using GraphPad Prism 5.0.

## Results

### Intracellular *T. cruzi* Infection Affects Host Cell Nuclear Compartment and Transcriptional-Splicing Machinery

**Figure 1:**
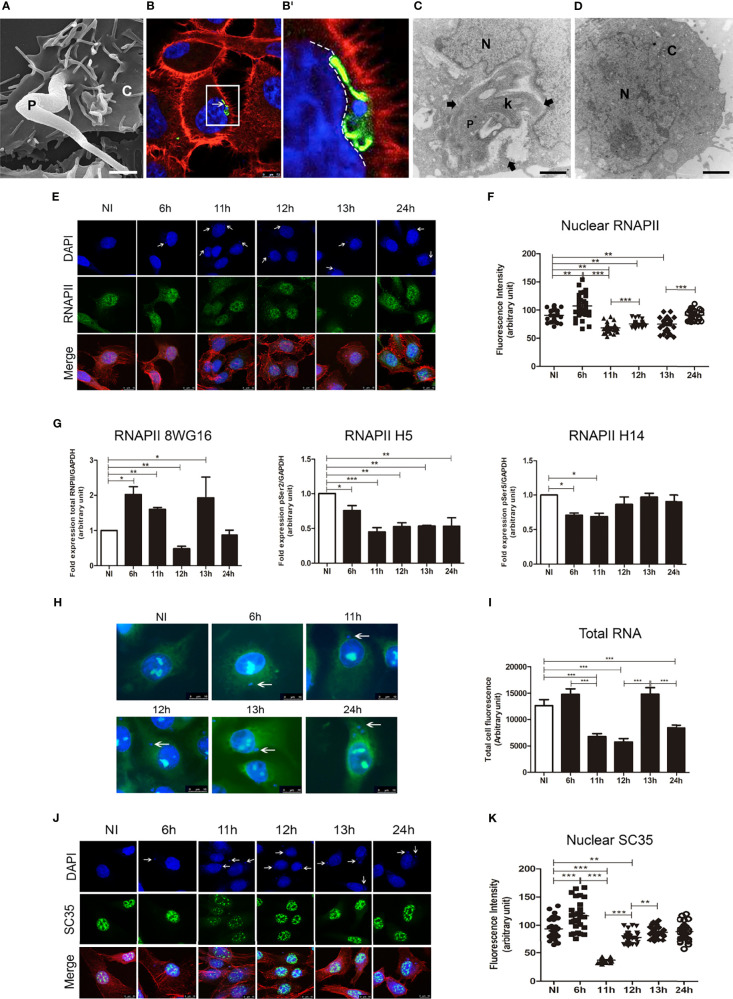
*Trypanosoma cruzi* infection can induce changes to the host nuclear compartment, affecting RNAPII and splicing machinery. **(A)** Host cell invasion mediated by trypomastigote form of *T. cruzi* observed by SEM showing the entry moment of the parasite (P) in the LLC-MK2 cell (C). Bar=2µm. **(B)** Confocal microscopy of LLC-MK2 cells after 4hpi with *T. cruzi* showing an intracellular trypomastigote form closest to the host nucleus (white arrow). Parasite flagellum is labeled with anti-TcFAZ antibody (green), host and parasite nuclei, and kinetoplast are stained with DAPI (blue). Host actin is stained with rhodamine-phalloidin (red). Bar=7.5µm. **(B’)**. Magnification corresponds to the inset in B (white box) showing the parasite causing a host nucleus deformation (white dashed line). **(C)** TEM image showing a longitudinal section of LLC-MK2 cell after 4hpi with *T. cruzi* emphasizing the juxtanuclear position of the parasite causing a NE deformation (black arrows). N=host nucleus, P=parasite, k=kinetoplast. Bar=1µm. **(D)** TEM image showing a longitudinal section of an uninfected LLC-MK2 (control). N=cell nucleus. C=cell cytoplasm. Bar=2µm. **(E)** Confocal microscopy of *T. cruzi* infected LLC-MK2 cells showing modulation of the RNAPII distribution at different times of infection (6–24hpi) and non-infected cells (NI) as a control. The total RNAPII is labeled with anti-RNAPII CTD (8WG16) domain antibody (green), actin is stained with rhodamine-phalloidin (red), and nuclei and kinetoplasts are stained with DAPI (blue). White arrows labeled intracellular parasites. Merged images are shown, as indicated. Bar=10µm. **(F)** Quantification of the nuclear RNAPII fluorescence intensity (n > 30 cells/time point) analyzed by ImageJ. **p < 0.005, ***p < 0.0001. One-way ANOVA (Bonferroni Multiple Comparison test; F=38.11; R2 = 0.5227). Data is representative of two independent experiments (n=2). **(G)** Fold expression of RNPII, RNAPII pSerine 2 and pSerine5, obtained from blotting images of *T. cruzi* infected LLC-MK2 (**Figure S1**), and quantified in ImageJ showing the protein modulations at different times of infection (6–24hpi) and NI cells as a control. Total RNAPII is labeled with anti-CTD (8WG16) domain antibody; *p < 0.05, **p < 0.005, One-way ANOVA (Tukey’s Multiple Comparison post-test; R^2^ = 0.69; F=5.278, p=0.009). Anti-H5 labels Serine 2 phosphorylation site; *p < 0.05, **p < 0.005, ***p < 0.0001 in One-way ANOVA (R^2^ = 0.82; F=9.725, p=0.007) and anti-H14 labels Serine 5 phosphorylation site; *p < 0.05 in One-way ANOVA (R^2^ = 0.48; F=3.349, p=0.026). Data were normalized by the amount of GAPDH protein. Statistics represent mean ± SEM of three independent experiments (n=3). **(H)** Total RNA of LLC-MK2 infected *T. cruzi* cells (6-24hpi) and non-infected (NI) cells are stained with Syto RNA (green) and visualized by Confocal microscopy. Host and parasite nuclei and kinetoplasts are stained with DAPI (blue). White arrows indicate the presence of the intracellular parasites. Bar=10µm. **(I)** Total RNA fluorescence intensity from confocal images (n > 30 cells/timepoint) were quantified and analyzed in ImageJ. ***p < 0.0001 (Bonferroni Multiple Comparison test; F=16.55; R^2^ = 0.3922). Data represent mean ± SEM of three independent experiments (n=3). **(J)** Confocal microscopy showing the distribution of nuclear speckles labeled with anti-SC35 antibody (green) in LLC-MK2 cells during (6–24hpi) with *T. cruzi* and non-infected cells (NI) as a control. Rhodamine phalloidin stained actin (red). Nuclei and kinetoplasts are stained with DAPI (blue). White arrows indicate intracellular parasites. Bar=10µm. **(K)** Analysis of the nuclear SC35 fluorescence intensity of images acquired during parasite progression of infection (n > 30 cells/timepoint). The quantifications were analyzed in ImageJ. Data is representative of two independent experiments (n=2). **p < 0.005, ***p < 0.0001. One-way ANOVA (Bonferroni Multiple Comparison post-test; F=80.95; R^2^ = 0.6994).

During the first stage of infection, trypomastigotes must interact with the host-surface molecules to adhere, penetrate, and transit into the host cell cytoplasm (Andrade and Andrews, 2005). Non-specialized phagocytic culture LLC-MK2 cells were infected with *T. cruzi* metacyclic trypomastigote forms as observed by Scanning Electron Microscopy to investigate this initial internalization (SEM: **Figure 1A**). During the first hours of *T. cruzi* infection (up to 2hpi), the internalized trypomastigote form is found within the host cytoplasm, visualized by flagella staining using an anti-TcFAZ antibody (in the house). Strikingly, we observed, in some cases, parasites localizing in close proximity to the host cell nucleus, correlating with deformation of the nuclear envelope (NE) (**Figures 1B, B’**), which we confirmed by Transmission Electron Microscopy (TEM; **Figure 1C**) when compared to non-infected (NI) cells (**Figure 1D**). NE alteration can impact chromatin distribution and consequently alter transcription regulation and splicing processes (Uhler and Shivashankar, 2017; Aureille et al., 2019; Huber et al., 2020). In all, our results suggest that infection with *T. cruzi* alters the host nuclear structure.

After *T. cruzi* invasion, early and late endosomes and lysosomes from the host endosomal-lysosomal compartment contribute to forming the nascent parasitophorous vacuole (PV), which encompasses the parasites (Andrade and Andrews, 2005). The ability of *T. cruzi* to interconvert between trypomastigote and amastigote forms is key for the pathogenesis of Chagas disease, and the transition between forms encompasses the disruption of the parasitophorous vacuole (PV). Prior reports show that PV disruption occurs at 12 to 15hpi, depending upon host cell type and parasite strain (Stecconi-Silva et al., 2003). At this time point, the parasites differentiate to replicative amastigote forms, which proliferate within the host cytoplasm (El-Sayed et al., 2005; Chiribao et al., 2014). Thus, to examine the effect on the host cell during the intracellular cycle of the parasite, we carried out our experiments at time points between 6–24hpi, focusing on time points within 11–13hpi, which are associated with the periods of PV rupture, when the parasites are released to the host cytoplasm, and the infection persists. Given that we observed NE deformation correlating with nuclear proximal *T. cruzi* parasites, we reasoned increased proximity of *T. cruzi* parasites to the host nucleus may reflect an as yet uncharacterized interaction between parasite and host cell. Recent data in human cells support the ability of the nucleus to sense, transduce and putatively alter gene expression in response to mechanical stimuli. With this in mind, we tested whether the NE deformation caused by the presence of *T. cruzi* could impact the localization or formation of active transcription foci in the nuclei of infected cells.

First, we tested for effects on host RNA polymerase II (RNAPII) by immunofluorescence (IF) using an antibody directed to the C-terminal domain (CTD - 8WG16). We observed a significant decrease in the fluorescence intensity of RNAPII nuclear foci between 11 and 13hpi within host cells infected with *T. cruzi* compared to control (NI) cells (**Figures 1E, F**). Western blot analysis of whole protein lysates, at the same time points used for IF, were performed using anti-CTD 8WG16 antibody, revealed a marked reduction in RNAPII protein levels at 12hpi, whose levels rapidly recovered at 13hpi (**Figures 1G** and **S1**). At 13hpi, we detected a signal equating to ~ two times the levels of the uninfected cells. This experiment was repeated more than three times with equivalent results.

The CTD of RNAPII subunit B1 (RPB1), the largest subunit of RNAPII, contains tandem heptapeptide repeats with the consensus sequence YSPTSPS. The phosphorylation of these residues during the transcription cycle, associated with a highly dynamic interaction network at the site of transcription that governs the quality of the transcribed RNA. The phosphorylation of the Serines 2 and 5 residues within CTD can influence the recruitment of specific factors that regulate the transcription elongation complex (Cheng and Sharp, 2003). Additionally, to investigate the effects on the phosphorylation of RNAPII, we used an anti-H5 antibody, which specifically recognizes phosphoserine 2 (PhosphoSer2) of the CTD domain of the RNAPII. Anti-H14 antibody was applied to label PhosphoSer5, which is linked to recruiting elongation factors and RNA processing proteins. We found that the levels of PhosphoSer 2 and 5 are reduced relative to the corresponding control sample until 13hpi of *T. cruzi* infection, with a marked decrease at 11hpi. Combined with the phosphorylation data, our results suggest an overall reduction in protein production and indicating RNAPII may reduce its transcriptional activity within an hour (**Figures 1G** and **S1**). Similar behavior of RNAPII largest subunit degradation without affecting the accumulation of its phosphorylated forms was described for influenza virus during infection (Rodriguez et al., 2007).

Next, we examined the total RNA in the infected LLC-MK2 cells compared to the non-infected cells (NI, control) using Syto RNA Select (Molecular Probes). The confocal analyses revealed both the levels of total RNA in the host cell and parasite during the infection (**Figure 1H**). By quantifying the total fluorescence area, we show a decrease in the total RNA abundance at 11hpi and 12hpi compared to NI cells (**Figure 1I**). However, at 13hpi, a significant increase in total RNA occurred, similar to the RNAPII results (**Figures 1G** and **S1**), suggesting the intriguing possibility that the host cell transcription machinery may alter in response to the intracellular cycle of the parasite.

Our data suggest altered transcription arises in host cells infected by *T. cruzi*. Given this, we next asked whether the nuclear speckles (NS, also known as interchromatin granule clusters), which are the nuclear sites for splicing factors storage, were affected by the presence of the parasite, since these organelles display considerable plasticity upon cellular stress (Carmo-Fonseca and Rino, 2011). The main component in speckles is SC35, which acts within transcriptional factories (Galganski et al., 2017). Using the SC35 splicing factor antibody, we performed confocal microscopy on infected and uninfected (NI) host cells. We found alterations in speckle distribution as the intracellular infection progressed (**Figures 1J, K**). When NS fluorescence intensity was quantified, we initially observed an increase in NS fluorescence intensity at 6hpi, which drastically decreased at 11hpi, and remained at the level of the uninfected cells up to 24hpi (**Figure 1K**). At 11hpi, the SC35 pattern changed to a more punctate distribution, as previously reported during an influenza virus infection (Fortes et al., 1995).

### Downregulation of the Host Heterogeneous Nuclear Ribonucleoproteins Is Mediated by Intracellular *Trypanosoma cruzi* in a Time-Dependent Manner

*Trypanosoma cruzi* infection is associated with alterations of splicing-related transcription (**Figure 1**). To deepen our understanding of the host nuclear activities during *T. cruzi* infection, we analyzed the behavior of the host hnRNPs (A1 and A2B1), which orchestrate a multitude of crucial cellular processes. Activities of hnRNP A1 relate to RNA metabolism, including gene expression regulation, RNA transport, and RNA stability (Jean-Philippe et al., 2013). A2B1 is associated with mRNA stability and cytoplasmic trafficking (Long and Caceres, 2009; Han et al., 2010).

**Figure 2:**
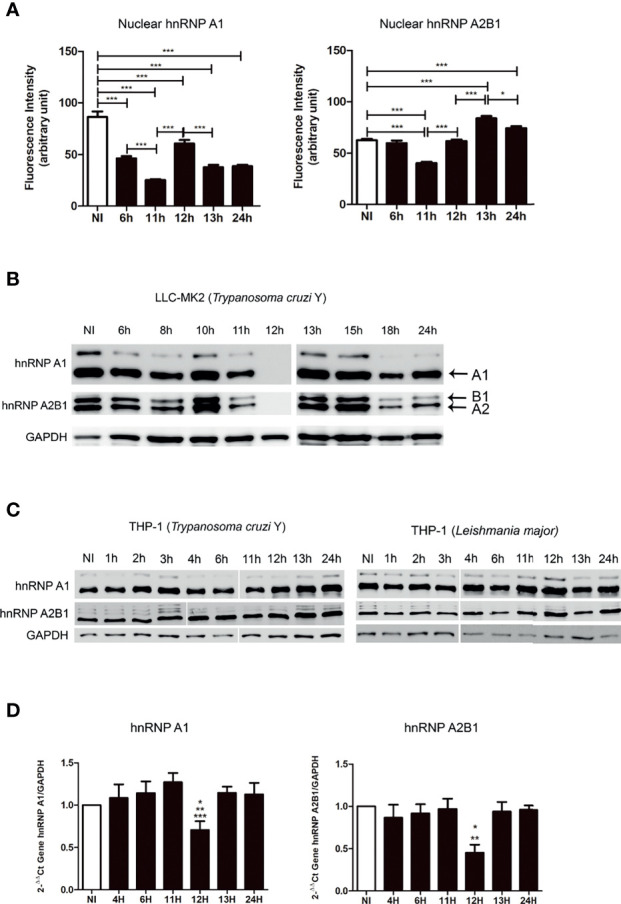
Downregulation of host hnRNPs in non-phagocytic cells infected with *T. cruzi*. **(A)** Quantifications of the nuclear fluorescence intensity by ImageJ software obtained from the LLC-MK2 cells infected (6–24hpi) with *T. cruzi* and non-infected (NI) cells as a control labeled with antibodies against hnRNPs (A1 and A2B1). The nuclear fluorescence intensity is quantified using images (n>30 cells/timepoint) obtained in the epifluorescence microscopy. *p<0.01; **p < 0.001; ***p<0.0001. One-way ANOVA (Bonferroni Multiple Comparison Test. For A1: F=52.40; R^2^ = 64.53; for A2B1: F=63.22; R^2^ = 0.6870). Data presented are mean ± SEM, representative of three independent experiments (n=3). **(B)** Western Blot of LLC-MK2 cells lysates highlighting hnRNPA1 and A2B1 levels at different times of infection (6-24hpi, NI–non-infected cells as a control) using anti-hnRNPs A1 and A2B1 antibodies, respectively, and indicated. GAPDH labeled with anti-GAPDH antibody is used as endogenous control. Data are representative of three independent experiments (n=3). **(C)** Western Blot of phagocytic THP-1 cell lysates infected with *T. cruzi* and with *L. major* (C’) in the early times of infection (1–24h and NIs) using anti-hnRNPs A1 and A2B1, respectively. GAPDH used as a loading control. Each experiment was done in biological triplicate (n=3). **(D)** Real-time qPCR analysis of mRNAs for hnRNPs (A1 and A2B1) in LLC-MK2 cells infected with *T. cruzi* at different times (6–24hpi) and NIs cells as a control. Data were normalized by the amount of GAPDH mRNA, expressed relative to the corresponding value for the cells, and are means ± SD from triplicate data (n=3) and were analyzed by SDS7500 software (Applied) using 2^-ΔΔCT^. *p < 0.01; **p < 0.001; ***p < 0.0001. One-way ANOVA (Tukey’s Multiple Comparison Test, p < 0.05).

Immunofluorescence analyses showed that host hnRNPs are predominantly localized in the nucleus, while A2B1 was also distributed in the nucleus and cytoplasm (**Figure S2**). The hnRNPs nuclear fluorescence intensity was quantified, and for hnRNP A1 we observed a strong decay of the nuclear fluorescence intensity at 6hpi, remaining below non-infected cell (control) levels (**Figure 2A**). In contrast, host nuclear A2B1 fluorescence intensity became reduced at 11hpi and recovered to levels comparable to that of control cells one hour later (12hpi). At 13hpi, the A2B1 signal remained higher than that of the control cells (**Figure 2A**). We also examined protein expression levels of these hnRNPs during parasitemia by Western Blot (**Figure 2B**). Strikingly, we found that all hnRNPs analyzed, which were probed for using protein-specific antibodies, were poorly detected at 12hpi, indicating a reduction of more than 98% compared to the non-infected cells (**Figures 2B** and **S3**). This decrease began at 11hpi, and within one hour, RNP levels became largely undetectable, whereas total protein expression levels were restored in the next hour (13hpi), similar behavior of RNAPII (**Figure 1G**).

To ask if these alterations on the behavior of hnRNP A1 during *T. cruzi* infection are cell line dependent, we tested our hypothesis in another cell line. HeLa is a non-specialized phagocytic epithelial cell line that is permissible to infection by *T. cruzi* (Maeda et al., 2012). We detected decreased expression of hnRNP A1 at 12hpi, in keeping with our data from LLC-MK2 cells, indicating a conserved mechanism occurring independently to host cell type (**Figure S4A**).

To ask if this effect was specific to our strain of *T. cruzi* or was a general effect of *T. cruzi* infection, we performed this analysis using another strain of *T. cruzi* - Tulahuen, a Chilean isolate (**Figure S4B**). Data generating using the Tulahuen strain revealed that hnRNP A1 expression disappeared at 11hpi and slowly returned at 15hpi, in contrast to the strain Y, where it disappeared at 12 hpi and recovered at 13 hpi (**Figure 2B**). Such differences could be explained by genetic differences between these strains as strain Y belongs to the TcII typing unit, and Tulahuen is classified as TcVI (Zingales et al., 2009). Besides the difference in their genetic markers, these two strains lead to different remodeling of the host cell transcriptome in infected myoblasts. Tulahuen strain has significant negative regulation of genes related to RNA splicing than in strain Y (Nisimura et al., 2020). This may be an indication of the reason for the decrease in hnRNP A1 in a longer time window in LLC-MK2 cells infected (**Figure 2B**) with the Tulahuen strain (**Figure S4B**).

We compared hnRNPs expression profiles from non-phagocytic cells (**Figures 2B** and **S2**) to expression profiles from THP-1 macrophages, which are professional phagocytic cells (**Figure 2C**). We observed that all hnRNPs (A1 and A2B1), which were not initially detected in non-phagocytic cell lines at 12hpi, were detected in *T. cruzi* infected THP-1 cells (**Figure 2C**). These results suggest a conserved mechanism in non-specialized phagocytic cells affected by *T. cruzi* infection. To complement this observation, we infected the THP-1 cells with *Leishmania major*, and a similar pattern of hnRNPs was observed as described for *T. cruzi* (**Figure 2C**).

Next, we examined whether the molecular mechanism underlying the hnRNPs (A1 and A2B1) correlates with the downregulation of their transcripts. We examined host hnRNPs A1 and A2B1 transcript levels using RT-qPCR, and at 12hpi, we found a decrease of ~30% and 65%, respectively, in agreement with the protein level reductions as shown in **Figure 2B** (**Figure 2D**). Our result suggests that *T. cruzi* can interfere in the activity of host proteins whose functions pertain to RNA processing.

### *Trypanosoma cruzi* Hijacks U2AF35 Affecting Host Cell Splicing Activity

The formation of the spliceosome complexes requires hnRNPs in conjunction with splicing proteins, including members of the serine- and arginine-rich (SR) family of proteins (U2AF, SC35) linked to RNAPII (Chathoth et al., 2014). So far, we have demonstrated that RNAPII CTD, total RNA, and splicing factor SC35 fluorescent levels decreased at 12h after host infection with *T. cruzi* (**Figure 1**), coinciding with a substantial down regulation of hnRNPs at the same time point (**Figure 2B**), indicating a reduction of host transcriptional activity.

**Figure 3:**
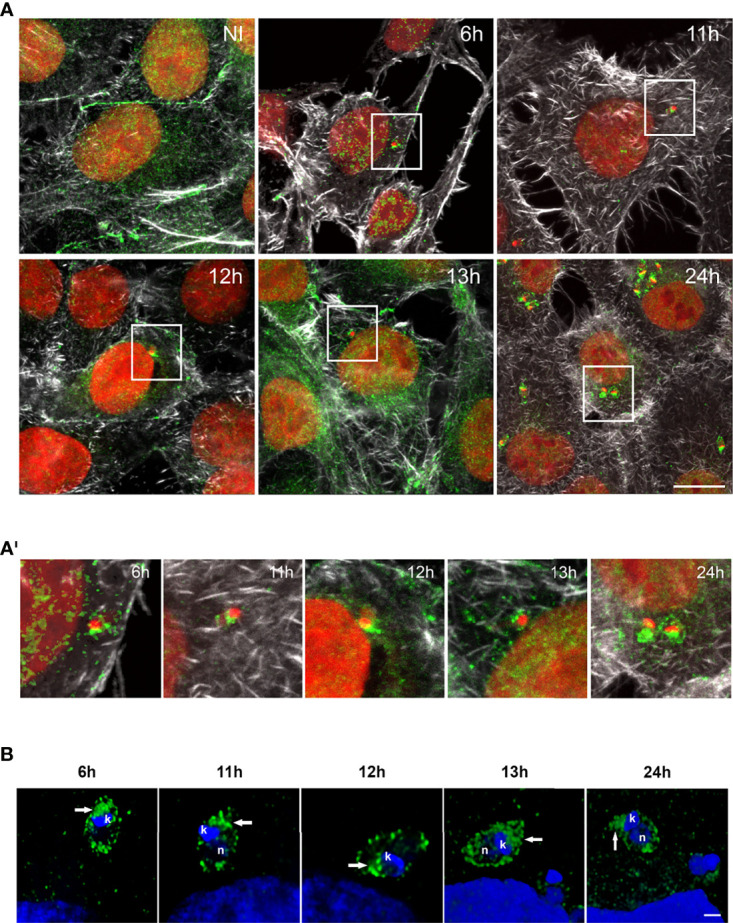
*Trypanosoma cruzi* hijacks host U2AF35 auxiliary splicing factor during cell infection. **(A)** Three-dimensional (3D) maximum intensity projection reconstruction from confocal images obtained at TCS-SP5 Leica Confocal Microscope showed the distribution of U2AF35 in LLC-MK2 infected (6–24hpi) and non-infected (NI) cells labeled with anti-U2AF35 antibody (green). Insets are highlighting the intracellular parasites (white boxes). Rhodamine phalloidin stains actin (grey). Host and parasite nuclei and kinetoplasts are stained with DAPI (red). **(A’)**. Enlarged area from insets in A (white boxes) showing the intracellular parasite and the localization of the host U2AF35 protein. Bar=10µm. **(B)** The localization and distribution of the host U2AF35 auxiliary splicing factor in the intracellular *T. cruzi* (6–24hpi) using anti-U2AF35 antibody (green) is analyzed by the Super Resolution confocal microscopy (LSM 880 with Airyscan super-resolution module- Carl Zeiss). White arrows are pointed out the flagellar pocket structure. Host and parasite nuclei and kinetoplasts are stained with DAPI (blue). N=parasite nucleus and k=kinetoplast. Bar=2µm.

To further examine the spliceosome formation, we investigated U2 snRNP auxiliary factor 1 (U2AF35), a 35kDa subunit, which is a heterodimer consisting of 35- and 65-kDa subunits (U2AF65 and U2AF35) in the processing of both constitutively and alternatively spliced pre-mRNAs (Voith Von Voithenberg et al., 2016). U2AF35, but not U2AF65, specifically facilitates branch point recognition, the formation of lariat introns (Kralovicova and Vorechovsky, 2017), and interactions with SR splicing factors (Wu and Maniatis, 1993). Using three-dimensional (3D) maximum intensity projection reconstructions of confocal microscopy images revealed that in non-infected cells (NI), the U2AF35 splicing factor is localized mainly in the host nucleus and finely dispersed in the cytoplasm (**Figure 3A**), in accordance with prior reports (Chusainow et al., 2005). However, after 6hpi with *T. cruzi*, the localization of U2AF35 extends from the nuclear area of the host cell to the cell body of the parasites, encompassing the entire corpus but excluded from their nuclei (**Figures 3A, B**). As the parasites proliferate, the U2AF35 signal accumulates within the cell body of the parasite and in the flagellar pocket near the kinetoplast (**Figures 3A, A’**, lower panels). We further confirmed the distribution of U2AF35 within the parasites by Super-Resolution microscopy images (**Figure 3B**). Western blotting analysis showed that the levels of the U2AF35 protein are also decreased at 12hpi during *T. cruzi* infection (**Figure S5A**), as we have demonstrated for other nuclear proteins in this work. Likewise, host U2AF35 transcript levels detected by RT-qPCR decreased at 12hpi, agreeing with the protein level reductions (**Figure S5B**).

The sequestering of the U2AF35 splicing factor has been previously reported during *Shigella* infection (Okuda et al., 2005). Also, the displacement of host nuclear proteins has been demonstrated during infection with the protozoan pathogen Theileria (Huber et al., 2020).

Using BLAST searches, we found no similarity between *T. cruzi* proteins to the mammalian U2AF35 sequence or the immunogenic sequence of the commercial U2AF35 antibody, observed in the TritrypDB alignment (**Figures S5C, D**). The U2AF35 splicing factor is also present in *T. cruzi* thus, to rule out cross-reactions between the commercial U2AF35 antibody and the *T. cruzi* factor, we performed IF and WB analysis on cultured trypomastigotes and axenic epimastigotes analyzed (**Figures S5A, E**). Our results show no cross-reactivity. Furthermore, though the large U2AF35 subunit is highly conserved in eukaryotes, it is divergent in trypanosomatids (Vázquez et al., 2003). In the trypanosome U2AF35 splicing factor, tryptophan 134, which is necessary for heterodimerization with U2AF65 in mammals, is mutated to lysine, suggesting that interaction between SF1-U2AF35-U2AF65 could be altered in trypanosomatids (Vázquez et al., 2003). It is noteworthy that other nuclear proteins analyzed in this work - RNAPII, SC35, hnRNP A1, and A2B1 - were not recruited by the parasite during infection (**Figures 1E, J**, **S2**).

**Figure 4:**
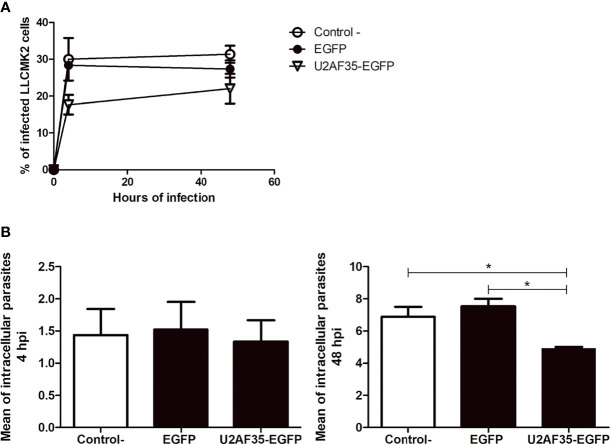
U2AF35-EGFP recombinant protein transient transfected in LLC-MK2 cells affect the number of intracellular amastigotes during infection. **(A)** Analysis of the percentage of infected cells under three different conditions: WT (Control -), transfected with empty-EGFP (EGFP) and transfected with U2AF35-EGFP. At least 100 cells/condition were analyzed in biological triplicated (n=3). **(B)** Analysis of the mean of intracellular parasite of LLC-MK2 cells transfected in three different conditions at 4hpi and 48hpi. ± SEM from the means of biological triplicate data (n=3) obtained from the counting of at least 100 cells/condition. *p < 0.05. One-way ANOVA (Tukey’s Multiple Comparison Test, p<0.05; F=9.171; R^2^ = 0.7535).

To better understand the role of the splicing factor U2AF35 during infection by *T. cruzi*, we investigated whether overexpression of U2AF35 in mammalian cells could affect infection and proliferation dynamics. LLC-MK2 cells were transfected with either U2AF35-EGFP or control empty vector (EGFP), then incubated with *T. cruzi* for 4h and 48h. Strikingly, transfection of U2AF35-EGFP significantly affects the capability of *T. cruzi* to infect and proliferate when compared to EGFP and to control cells (**Figure 4A**). Furthermore, at 48hpi, the growth rates of intracellular amastigotes decreased with overexpression of host U2AF35 splicing factor hindering the survival and persistence of *T. cruzi* within host cells (**Figure 4B**).

**Figure 5:**
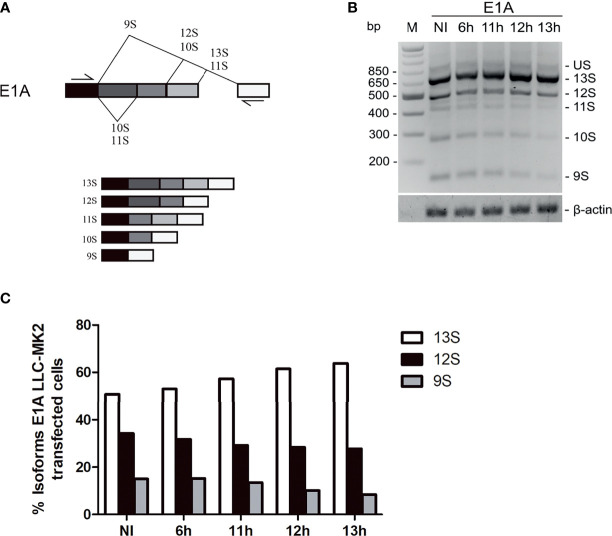
*Trypanosoma cruz*i regulates E1A alternative splicing in LLC-MK2 cells. **(A)** Schematic representation of the E1A pre-mRNA showing the E1A-derived splicing variants: Unspliced RNA (US) and splicing products (13S, 12S, 11S, 10S and 9S). **(B)** Splicing of E1A was amplified by PCR in E1A minigene-transfected cells, following infection with *T. cruzi* at different times of infection (6–13hpi) and NI. Splicing products are analyzed in 3% agarose gel and stained with ethidium bromide. DNA ladder is indicated on the left and splicing products are indicated on the right of the panel. β-actin gene was used as a loading control. **(C)** Quantification of major E1A mRNA isoforms (13S, 12S, and 9 S) from the data in B using β-actin to normalization; bars represent the percentage of each isoform variant at different times of infection and non-infected cells. Data is representative of one of the three independent experiments (n=3).

Next, to investigate whether the presence of *T. cruzi* could affect the regulation of pre-mRNA splicing, we carried out an *in vivo* splicing reporter assay. We transfected an adenovirus E1A minigene reporter (Cáceres et al., 1994) into LLC-MK2 cells for 24h and afterward infected the cells with *T. cruzi* at varying times (6–13h and NI as control). When processed by the spliceosome complex, the E1A splicing assay generates multiple mRNA splicing forms (13S, 12S, 11S, 10S, and 9S) when processed by the spliceosome complex (**Figure 5A**). During infection progression, intracellular *T. cruzi* shows its role in maturing E1A spliced products, tipping the balance towards less alternative spliced forms as infection progressed (**Figures 5B, C**). The 9S splicing isoform, the processed mRNA, with the most distal E1A alternative 5′ splice site, presents ~50% reduction after 12hpi compared to NI. In conjunction, the intermediate form (13S) levels increased to ~10% (**Figure 5B, C**), suggesting *T. cruzi* can interfere with the host splicing machinery.

**Figure 6:**
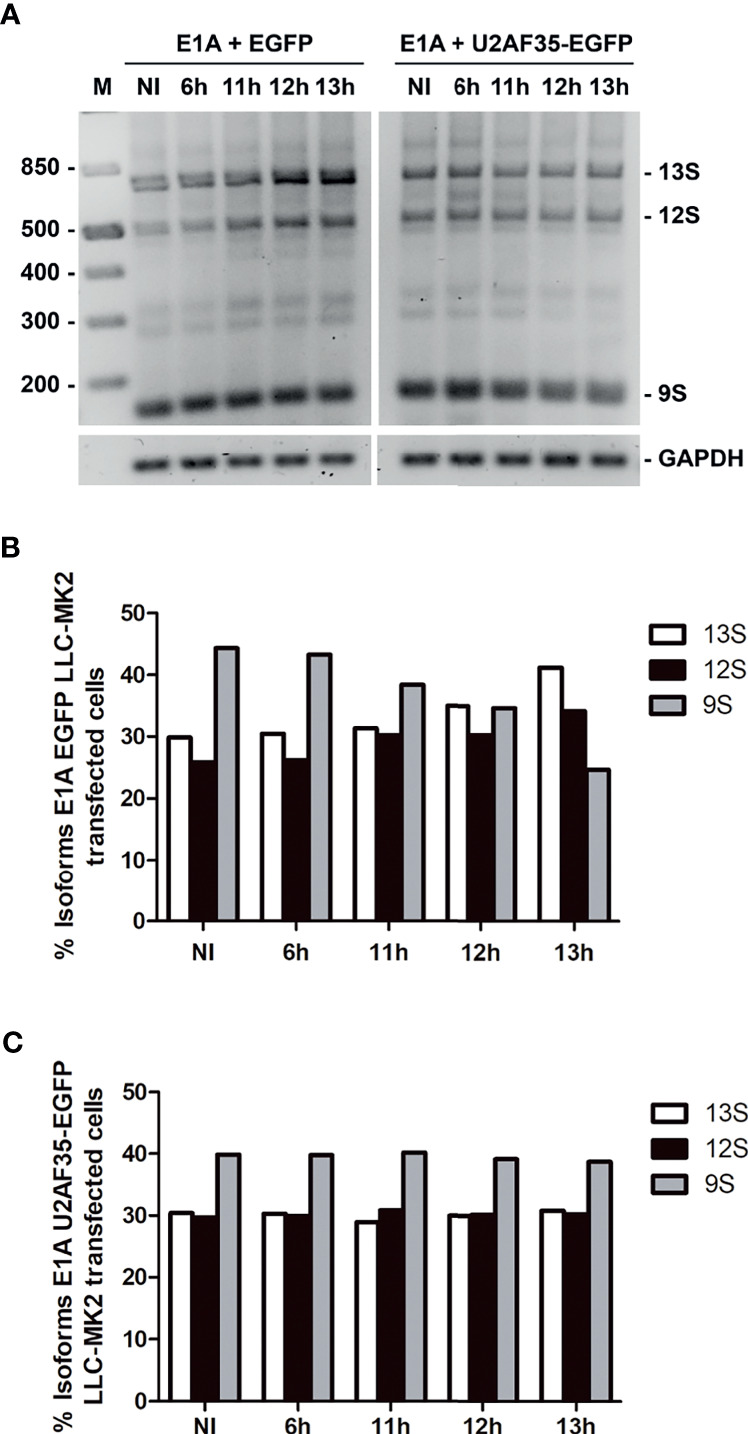
Addiction of exogenous U2AF35-EGFP recovered the E1A alternative splicing in LLC-MK2 infected *T. cruzi* cells. **(A)** Splicing of E1A was amplified by PCR in cells co-transfected with E1A and U2AF35-EGFP after infection with *T. cruzi* at different times (6–13hpi) and non-infected cells (NI). LLC-MK2 cells co-transfected with the minigene reporter E1A and EGFP posteriorly infected were used as control. Splicing products are analyzed in 3% agarose gel and stained with ethidium bromide. DNA ladder is indicated on the left, and splicing products are indicated on the right of the panel. GAPDH gene was used as a loading control. **(B)** Quantification of major E1A mRNA isoforms (13S, 12S, and 9S) calculated from the data in **(A)** (E1A co-transfected with EGFP); bars represent the percentage of each isoform variant at different times of infection respectively and non-infected cells. The data normalization was made using GAPDH gene band. **(C)** Quantification of major E1A mRNA isoforms (13S, 12S, and 9S) calculated from the data in **(A)** (E1A co-transfected with U2AF35-EGFP); bars represent the percentage of each isoform variant at different times of infection and non-infected cells. The data normalization was made using GAPDH gene band. Data is representative of one of two independent experiments (n=2).

Additionally, we ask if the exogenous U2AF35 could favorably alter the E1A pre-mRNA splicing pattern since the overexpression of this auxiliary factor interferes with the parasite proliferation (**Figure 4**). We co-transfected LLC-MK2 cells with E1A and U2AF35-EGFP plasmids or an empty EGFP vector as a control (**Figure 6**). We observed that E1A + EGFP showed an increase in the proximal isoform 13S and a decrease in the levels of distal isoform 9S during the infection progression (**Figures 6A, B**), as demonstrated with E1A only (**Figure 5C**). Indeed, 5’ splice site inhibition was not observed when the E1A splicing reporter was co-transfected with U2AF35-EGFP. Our result shows a similar percentage of the three isoforms (13S, 12S, and 9S) during the analyzed times of infection (**Figure 6C**). In all, we have shown, for the first time, that intracellular *T. cruzi* directly affects spliceosome formation throughout hijacking an auxiliary splicing factor.

## Discussion

Herein, we show that intracellular *T. cruzi* localization outside the host nuclear envelope is linked to downregulation of the host cell transcription and splicing machinery in non-phagocytic cells. We propose that the nuclear deformation induced by the presence of the parasite in the host cell cytoplasm could promote chromatin remodeling, explaining the reduced transcription activity and changes in the host total RNA expression. These dynamic changes could influence downstream RNA processing events, including alternative splicing regulation, and may reflect an important mechanism by which *T. cruzi* can persist within a host cell. It is important to emphasize that the transcription and splicing changes were analyzed in the period surrounding the PV disruption (11-13hpi), which has never been shown before.

Chagas disease is a significant cause of morbidity and mortality in humans in Central and South America, leading to adverse health outcomes and a considerable economic impact (Pérez-Molina and Molina, 2018; Drugs for Neglected Disease initiative, 2021). This scenario sheds light on the need to understand the host-pathogen interaction better. Many of the mechanisms developed by parasites used to evade or manipulate the host immune response and establish infection remain vastly unknown.

We show here that during the first hours of infection, *T. cruzi* is located very close to the host cell nucleus, causing a NE morphology deformation (**Figure 1**), and such proximity to the nucleus could promote alterations in cell transcription and splicing. In fact, NE deformation has been observed during cancer cell migration (Denais et al., 2016), infection by Epstein-Barr virus (Lee et al., 2012), by *L. major* infection (Isnard et al., 2015) and Theileria protozoa (Huber et al., 2020). Physical stress caused by NE deformation can lead to a loss of nuclear integrity, resulting in DNA damage (Denais et al., 2016; Raab et al., 2016). Also, external mechanical forces can remodel the NE, affecting nuclear structure and function (Aureille et al., 2017). Moreover, it has been described that protozoan parasite could manipulate host cells *via* epigenetic modifications, altering transcription and corresponding signaling pathways (Robert Mcmaster et al., 2016).

We found that RNAPII is rapidly dephosphorylated at 6 to 11hpi, displaying a marked reduction of the total protein level at 12hpi. It has been described that influenza virus polymerase associates with host RNA polymerase II (RNAP II) and causes specific degradation of the hypophosphorylated form without affecting the accumulation of its hyperphosphorylated forms. The inhibition should affect especially the transcriptional elongation and splicing activity (Rodriguez et al., 2007). Recent data reinforce our hypothesis showing a significant reduction in the newly transcripts synthesis during *T. cruzi* infection (Florentino et al., 2021).

The spatial proximity of nuclear speckles and active RNAPII transcription sites allows appropriate access to splicing factors. Splicing factors are continuously added to and released from RNAPII as this enzyme moves along a gene. The inhibition of either RNAPII transcription activity or splicing leads to the accumulation of splicing factor proteins into nuclear speckles (Galganski et al., 2017). In our data, SC35 splicing factor accumulated within NS at 6hpi, suggesting an inhibition of the transcription machinery at this time, followed by a considerable decrease in signal at 11hpi (**Figure 1**). Previous studies indicate that SC35 depletion induces RNAPII accumulation and attenuates elongation (Lin et al., 2008), thus the presence of *T. cruzi* could induce downregulation of host splicing factors which correlates with a reduction of RNAPII activity, resulting in a slow exon inclusion or exon skipping (Alexander et al., 2010; Chathoth et al., 2014; Simões et al., 2015; Jung et al., 2020).

Our results revealed that hnRNPs A1 and A2B1 are drastically downregulated in only one hour of parasite proliferation, from 11 to 12hpi, and return to normal levels at 13hpi, compared to uninfected LLC-MK2 cells (**Figures 2** and **S2**). These results suggest the existence of a mechanism where the presence of the parasite could affect the alternative splicing regulation at this point time. These hnRNPs are a conserved family of nuclear proteins that play diverse cellular roles, including the association with nascent RNAPII transcripts, and are known to be RNA binding proteins to modulate function (Graveley, 2000; Gama-Carvalho and Carmo-Fonseca, 2001; Long and Caceres, 2009; Han et al., 2010). The reduction of more than 98% in protein levels observed for hnRNP A1 and A2B1 are consistent with a significant drop in their transcription levels (**Figure 2**).

The regulation of alternative pre-mRNA splicing by hnRNP A1 and splicing factor SF2 are essential to precisely determining the selection of the 5’ splice site (Mayeda and Krainer, 1992). Additionally, SR proteins can induce the spliceosome formation, promoting interaction among U1 snRNP, U2AF complex, and the new RNA transcript, acting as an antagonist of hnRNPA1 (Zhu et al., 2001). During pre-mRNA maturation, the splicing factor SF1 is recruited and binds to a specific adenine located on the intron, recognizing the branch point sequence at 3’splice site (Voith Von Voithenberg et al., 2016). Then, U2AF35 serves as the auxiliary factor responsible for forming a complex with U2AF65 by establishing a stable interaction between U2 snRNA and the 3’ region of pre-mRNA intron during the initiation of spliceosome recruitment (Pacheco et al., 2004).

Our findings have several important implications for the field. First, *T. cruzi* is capable of hijacking host factors, in this case, the U2AF35 splicing factor meaning this parasite may developed this ability to adapt the host cell environment to better suit their proliferative needs. Secondly, intracellular *T. cruzi* can alter host cell processes, and here, our data suggest *T. cruzi* is capable of disrupting host cell spliceosome formation, and this might represent a major strategy of the parasite to subvert the host cell defense responses. Further investigations must be done to complement these studies.

Prior transcriptome analyses of cells infected with *T. cruzi* found evidence for altered transcription regulation as a result of the interaction between *T. cruzi* and its host cell (Jung et al., 2020; Houston-Ludlam et al., 2016; Li et al., 2016), though further experimental validation is required to strengthen these findings (Jung et al., 2020). Indeed, our findings are in keeping with this proposal. We demonstrated that the E1A alternative splicing pattern was altered in cells infected with *T. cruzi* compared to NI. Since the protein levels of the hnRNP A1 and U2AF35 were reduced in infected cells, an altered ratio of the alternative splicing is expected due to limits on the splicing factors available. The imbalance of the E1A proximal and distal isoforms was recovered with the presence of the exogenous U2AF35 during the analyzed infection times (**Figure 6**), emphasizing the importance of the sequestered U2AF35 factor for the continuity of the parasite infection (**Figures 4**, **6**). These results are the most direct evidence for the U2AF35 factor as an anti-parasitic strategy employed by the host cell.

Together, our results highlight the complexity of the host-pathogen interaction, adding a novel layer. More specifically, our data reflect a previously unappreciated capacity for *T. cruzi* to manipulate the host cell environment by interfering the dynamics of the host nuclear compartment in non-specialized phagocytic cells during infection in a time-dependent manner. Delineating these interactions further will likely reveal additional host factors targeted, and potentially usurped by these parasites thereby offering increased opportunities for the discovery of parasite specific pathways required for their intracellular cycle, and thus novel chemotherapeutic targets.

## Data Availability Statement

The original contributions presented in the study are included in the article/**Supplementary Material**. Further inquiries can be directed to the corresponding author.

## Ethics Statement

The animal study was reviewed and approved by Approved by CETEA (Comissão de Ética em Experimentação Animal da Faculdade de Medicina de Ribeirão Preto, approved protocol number 121/2016). CETEA follow the Brazilian national guidelines recommended by CONCEA (Conselho Nacional de Controle em Experimentação o Animal).

## Author Contributions

CG-C carried out the majority of the experiments. FF-C designed several primers, and performed DNA constructions and perform some PCR assays. CF carried out electron microscopy experiments. CF and FF-C carried out RT-qPCR analysis. HI-A carried out super-resolution microscopy and reviews the manuscript. RG-C carried out some WB experiments. CG-C, MG, and MB designed experiments, interpreted the results. CG-C and MB wrote the paper. All authors contributed to the article and approved the submitted version.

## Funding

This work was supported by Fundação de Amparo à Pesquisa do Estado de São Paulo (FAPESP 2010/19547-1; 2018/03677-5) to MB, (FAPESP 2018/01308-2) to MDG, by Fundação de Apoio ao Ensino, Pesquisa e Assistência- FAEPA to MB and by Coordenação de Aperfeiçoamento de Pessoal de Nível Superior- Brasil (CAPES) – finance code 001. CG-C received a master and doctoral fellowship from CAPES; RG-C received a master fellowship from CAPES.

## Conflict of Interest

The authors declare that the research was conducted in the absence of any commercial or financial relationships that could be construed as a potential conflict of interest.

## Publisher’s Note

All claims expressed in this article are solely those of the authors and do not necessarily represent those of their affiliated organizations, or those of the publisher, the editors and the reviewers. Any product that may be evaluated in this article, or claim that may be made by its manufacturer, is not guaranteed or endorsed by the publisher.

## Supplementary Material

Supplementary Figure 1 Host RNAPII is modulated during *T. cruzi* infection. Western Blot of *T. cruzi* infected LLC-MK2 cells showing the RNAPII modulations at different times of infection (6–24hpi) and NI (non-infected) cells as a control. Total RNAPII is labeled with anti-CTD (8WG16) domain antibody; lower blots are labeled with anti-RNAPII (H5, Serine 2 phosphorylation site and H14, Serine 5 phosphorylation site) and GAPDH is labeled with anti-GAPDH antibody and used as a loading control.

Supplementary Figure 2 Intracellular *T. cruzi* affects the distribution of hnRNPs in LLC-MK2 infected cells. Confocal microscopy shows the localizations of hnRNPA1 and A2B1 in LLC-MK2 cells at different times of *T. cruzi* infection (6-24hpi) and in non-infected cells (NI) as a control. HnRNPs were labeled with specific anti-hnRNPs (A1 and A2B1) antibodies (green) as indicated. Host and parasite nuclei and kinetoplasts are stained with DAPI (blue). Host actin is stained with rhodamine-phalloidin (red). Merged images are shown as indicated. White arrows labeled intracellular parasites. These results are representative of three independent experiments (n=3). Bars=10µm.

Supplementary Figure 3 Host hnRNP A1 and A2B1 are modulated during *T. cruzi* infection. Fold expression of hnRNP A1 and A2B1 from the blotting images (). Quantifications were done using ImageJ software and the normalization was made in relation to GAPDH protein band used as a loading control.

Supplementary Figure 4 The hnRNP A1 protein levels are downregulated in HeLa and in LLC-MK2 cells infected with *T. cruzi* Tulahuen strain. **(A)**. Western Blot of HeLa cells infected at different times (6-24hpi) with *T. cruzi* and NI (non-infected) cells as a control shows the distribution of the hnRNP A1. **(B)**. Western Blot of LLC-MK2 cells infected *T. cruzi* (Tulahuen strain) shows the hnRNP A1 protein levels at different times of infection (6-24hpi) and at NI cells as a control. GAPDH was used as a loading control in both experiments **(A, B)**. These results **(A, B)** are representative of two independent experiments (n=2).

Supplementary Figure 5 Anti-U2AF1 antibody immunogenic sequence present low similarity to the trypanosomatid amino acid sequences. **(A)** Complete amino acid sequence of Human U2AF35 showing in bold the immunogenic sequence (aa 60-93) used to produce the antibody in rabbit. **(B)** Alignment of Human U2AF35 immunogenic sequence (aa 60-93) against trypanosomatid genome bank using Blastp in the TriTrypsDB platform showing low significance of similarity among the proteins and the immunogenic sequence emphasizing the low SCORE and E (e-value - red box). **(C)** Western Blot using anti-U2AF35 antibody does not recognize free *T. cruzi* trypomastigote (Trypo) and nor epimastigote (Epi) forms. Non-infected LLC-MK2 cells (NI) and GAPDH are controls and ponceau stained is demonstrated the protein loading. **(D)** Confocal microscopy of free trypomastigote and epimastigote forms demonstrated the nuclei is not labeling with anti-U2AF35 antibody (green). Nuclei (N) and kinetoplasts (k) are stained with DAPI (blue). Phase-contrast merged images are shown as indicated. Bars=5µm (trypomastigote) and 3µm (epimastigote). **(E)** Real-time PCR analysis of U2AF35 mRNA from LLC-MK2 cells infected with *T. cruzi* at different times (4-24hpi) and non-infected cells (NI, control). Data were normalized by GAPDH mRNA (internal control), expressed relative to the corresponding value for analyzed times. Means ± SD of data in triplicate (n=3) and were analyzed by SDS7500 software (Applied) using 2 -ΔΔCT. *p<0.01; **p <0.001. One-way ANOVA (Tukey’s Multiple Comparison Test, p < 0.05).
