# Supplementary figures and images for: Modulation of the Host Nuclear Compartment by Trypanosoma cruzi Uncovers Effects on Host Transcription and Splicing Machinery
Source: Front Cell Infect Microbiol. 2021 Oct 19;11:718028. doi: 10.3389/fcimb.2021.718028 (PMC8560699; doi:10.3389/fcimb.2021.718028)

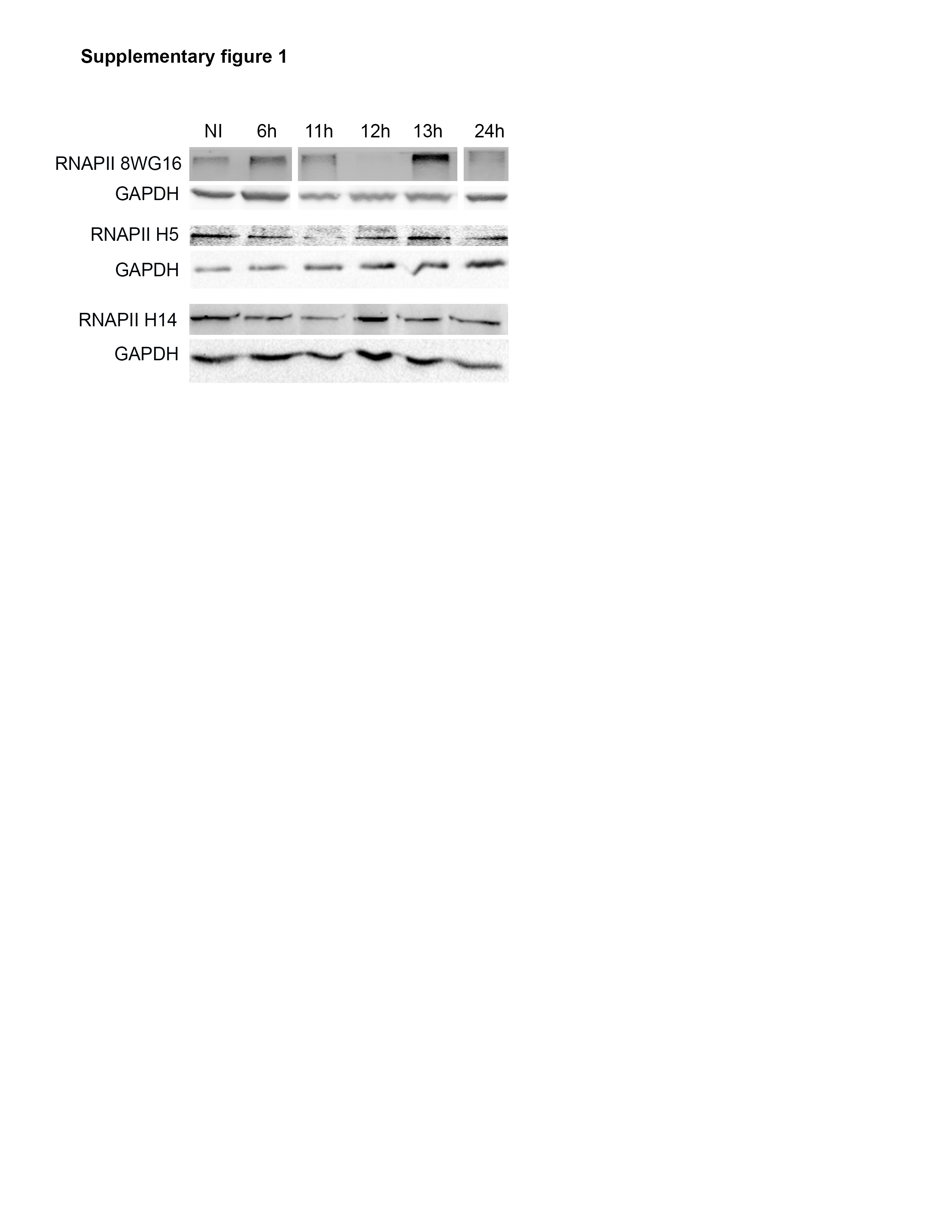

Supplement: Supplementary Figure 1 — Host RNAPII is modulated during T. cruzi infection. Western Blot of T. cruzi infected LLC-MK2 cells showing the RNAPII modulations at different times of infection (6–24hpi) and NI (non-infected) cells as a control. Total RNAPII is labeled with anti-CTD (8WG16) domain antibody; lower blots are labeled with anti-RNAPII (H5, Serine 2 phosphorylation site and H14, Serine 5 phosphorylation site) and GAPDH is labeled with anti-GAPDH antibody and used as a loading control. [file Image_1.tif]

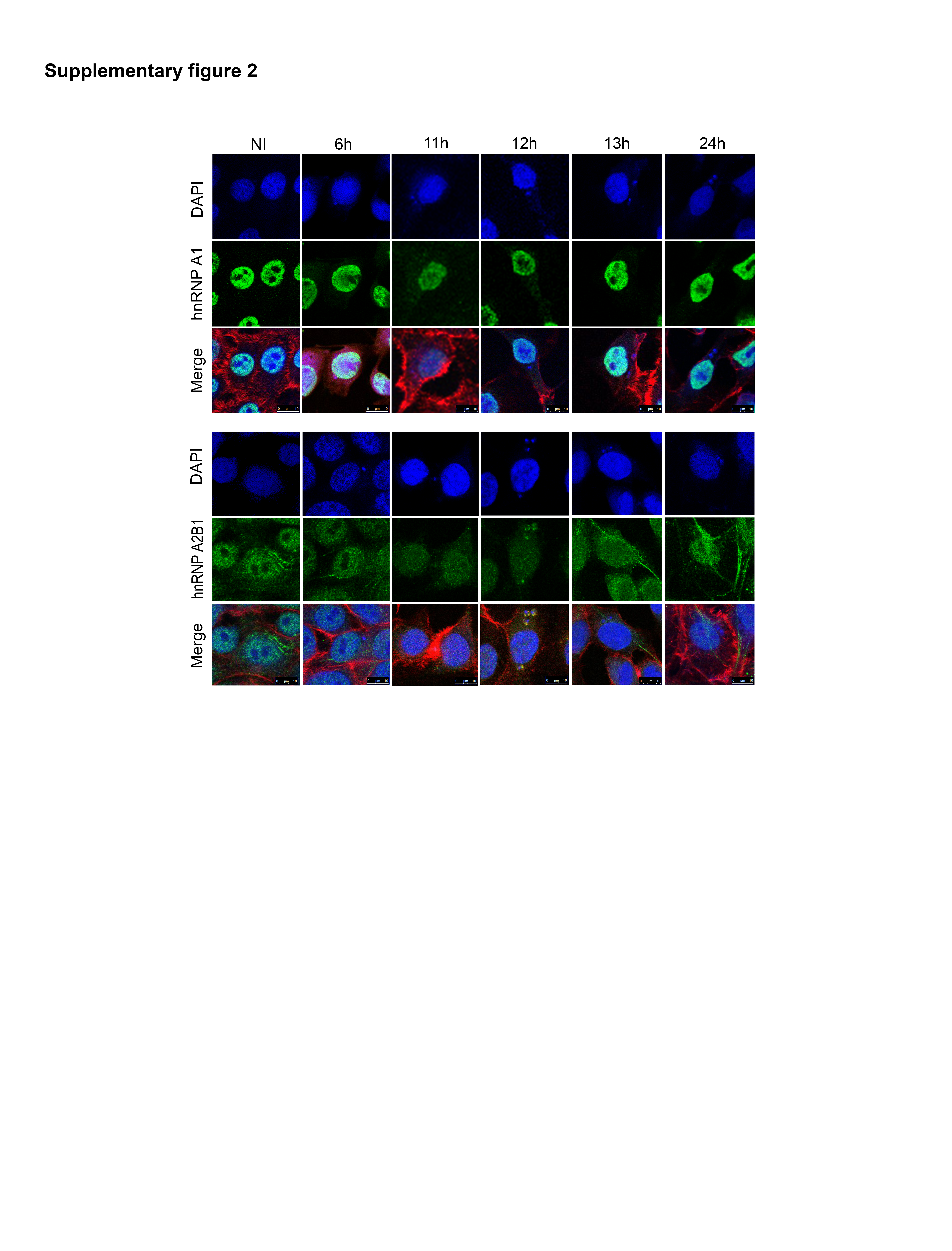

Supplement: Supplementary Figure 2 — Intracellular T. cruzi affects the distribution of hnRNPs in LLC-MK2 infected cells. Confocal microscopy shows the localizations of hnRNPA1 and A2B1 in LLC-MK2 cells at different times of T. cruzi infection (6-24hpi) and in non-infected cells (NI) as a control. HnRNPs were labeled with specific anti-hnRNPs (A1 and A2B1) antibodies (green) as indicated. Host and parasite nuclei and kinetoplasts are stained with DAPI (blue). Host actin is stained with rhodamine-phalloidin (red). Merged images are shown as indicated. White arrows labeled intracellular parasites. These results are representative of three independent experiments (n=3). Bars=10µm. [file Image_2.tif]

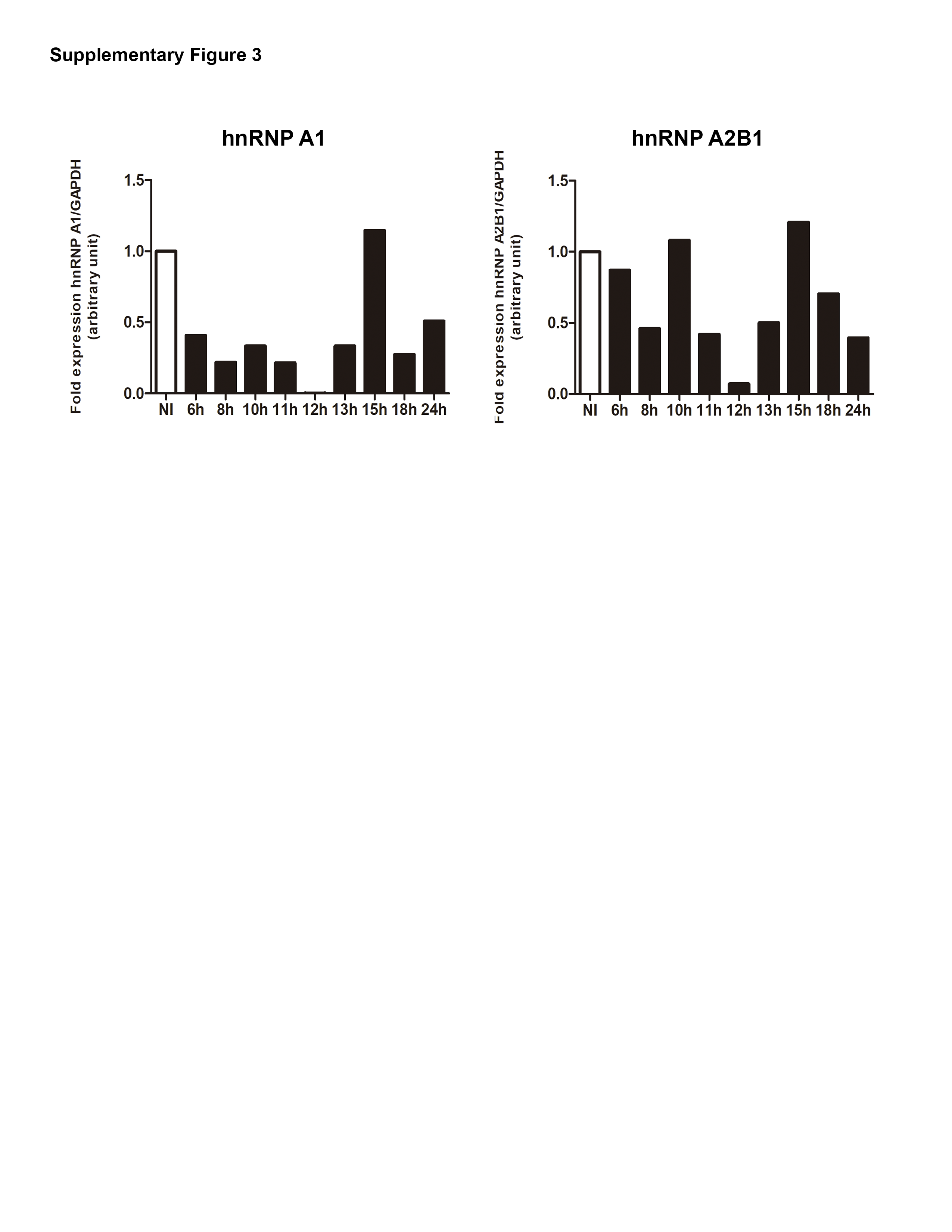

Supplement: Supplementary Figure 3 — Host hnRNP A1 and A2B1 are modulated during T. cruzi infection. Fold expression of hnRNP A1 and A2B1 from the blotting images (). Quantifications were done using ImageJ software and the normalization was made in relation to GAPDH protein band used as a loading control. [file Image_3.tif]

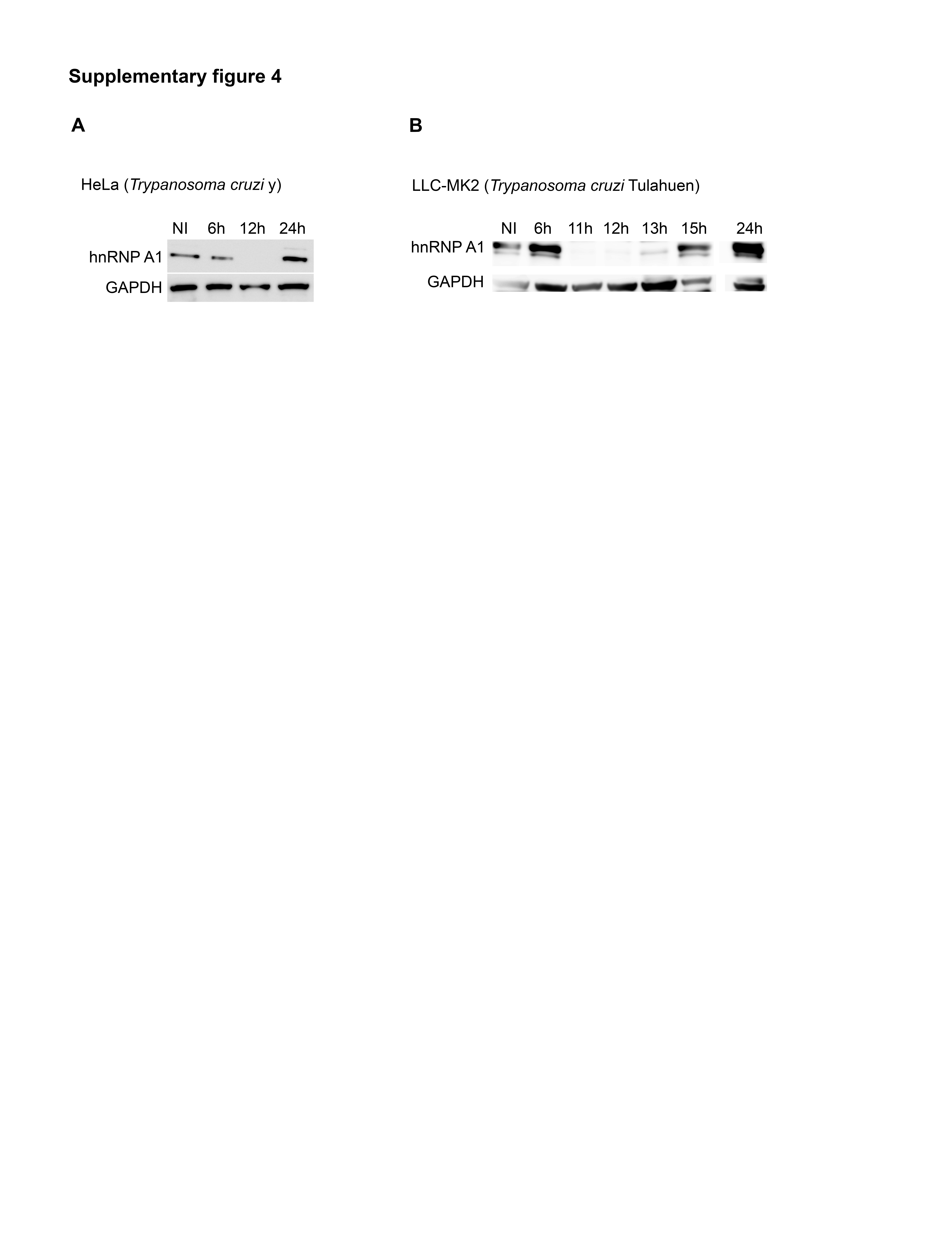

Supplement: Supplementary Figure 4 — The hnRNP A1 protein levels are downregulated in HeLa and in LLC-MK2 cells infected with T. cruzi Tulahuen strain. (A). Western Blot of HeLa cells infected at different times (6-24hpi) with T. cruzi and NI (non-infected) cells as a control shows the distribution of the hnRNP A1. (B). Western Blot of LLC-MK2 cells infected T. cruzi (Tulahuen strain) shows the hnRNP A1 protein levels at different times of infection (6-24hpi) and at NI cells as a control. GAPDH was used as a loading control in both experiments (A, B). These results (A, B) are representative of two independent experiments (n=2). [file Image_4.tif]

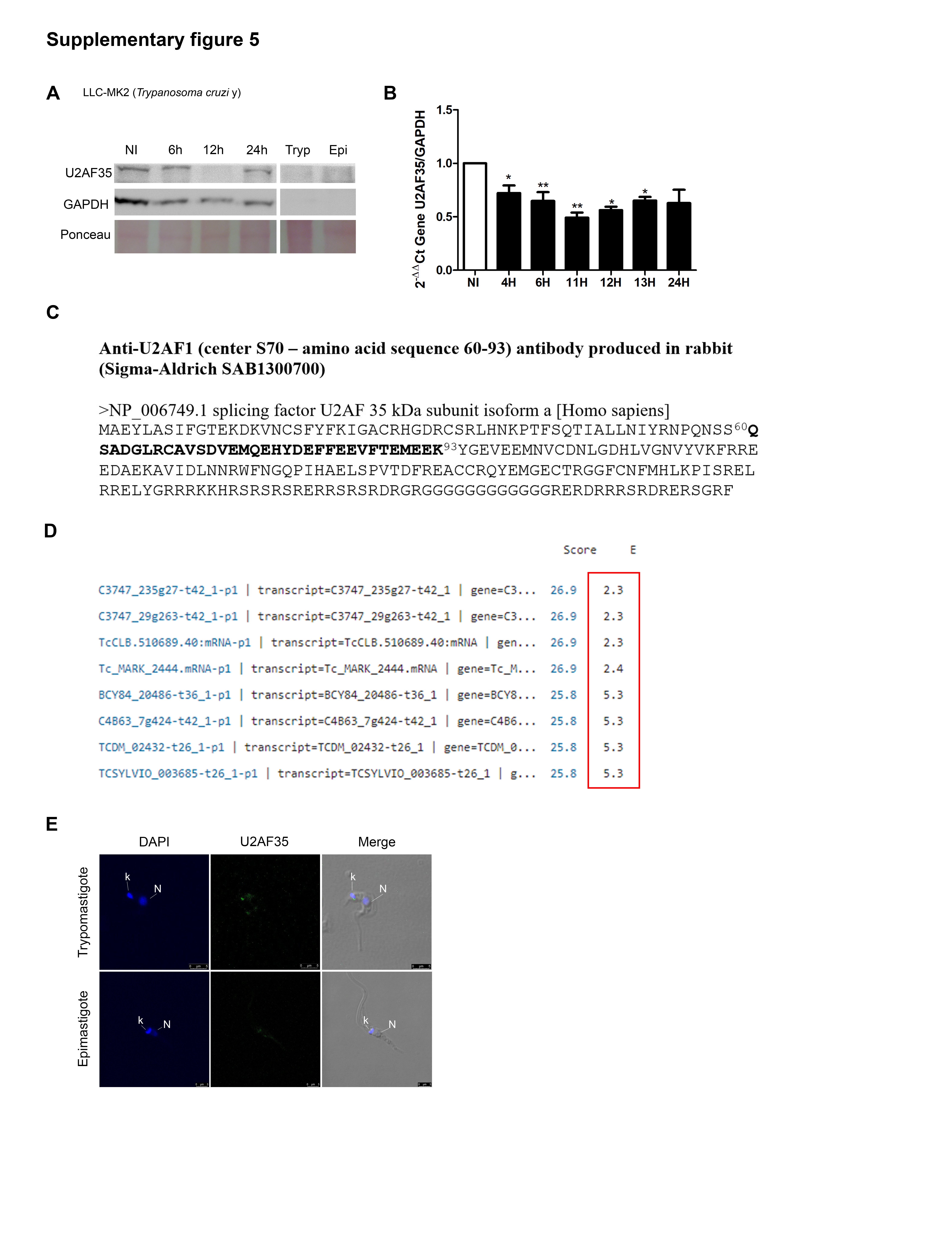

Supplement: Supplementary Figure 5 — Anti-U2AF1 antibody immunogenic sequence present low similarity to the trypanosomatid amino acid sequences. (A) Complete amino acid sequence of Human U2AF35 showing in bold the immunogenic sequence (aa 60-93) used to produce the antibody in rabbit. (B) Alignment of Human U2AF35 immunogenic sequence (aa 60-93) against trypanosomatid genome bank using Blastp in the TriTrypsDB platform showing low significance of similarity among the proteins and the immunogenic sequence emphasizing the low SCORE and E (e-value - red box). (C) Western Blot using anti-U2AF35 antibody does not recognize free T. cruzi trypomastigote (Trypo) and nor epimastigote (Epi) forms. Non-infected LLC-MK2 cells (NI) and GAPDH are controls and ponceau stained is demonstrated the protein loading. (D) Confocal microscopy of free trypomastigote and epimastigote forms demonstrated the nuclei is not labeling with anti-U2AF35 antibody (green). Nuclei (N) and kinetoplasts (k) are stained with DAPI (blue). Phase-contrast merged images are shown as indicated. Bars=5µm (trypomastigote) and 3µm (epimastigote). (E) Real-time PCR analysis of U2AF35 mRNA from LLC-MK2 cells infected with T. cruzi at different times (4-24hpi) and non-infected cells (NI, control). Data were normalized by GAPDH mRNA (internal control), expressed relative to the corresponding value for analyzed times. Means ± SD of data in triplicate (n=3) and were analyzed by SDS7500 software (Applied) using 2 -ΔΔCT. *p<0.01; **p <0.001. One-way ANOVA (Tukey’s Multiple Comparison Test, p < 0.05). [file Image_5.tif]
